# Supplementary material for: Cumulative effect of metabolic syndrome on the risk of retinal vein occlusion in young patients: A nationwide population-based study
Source: PLoS One. 2024 May 20;19(5):e0303871. doi: 10.1371/journal.pone.0303871 (PMC11104591; doi:10.1371/journal.pone.0303871)
Supplement: S1 Checklist — (DOCX) [file pone.0303871.s001.docx]

STROBE Statement—checklist of items that should be included in reports of observational studies

|  | Item No. | Recommendation | Page  No. | Relevant text from manuscript |
| --- | --- | --- | --- | --- |
| **Title and abstract** | 1 | (*a*) Indicate the study’s design with a commonly used term in the title or the abstract | 1 | Cumulative effect of metabolic syndrome on the risk of retinal vein occlusion in young patients: a nationwide population-based study |
|  |  | (*b*) Provide in the abstract an informative and balanced summary of what was done and what was found | 2 | This study aimed to investigate the impact of the cumulative burden of metabolic syndrome (MetS) on the incidence of retinal vein occlusion (RVO) in young adults. The risk of RVO was positively correlated with the cumulative number of MetS diagnoses over the four health examinations. All five MetS components were independently associated with an increased risk of RVO. Subgroup analysis for the impact of MetS on RVO occurrence revealed that MetS had a greater impact on female subjects (P <0.001). |
| Introduction | | | |  |
| Background/rationale | 2 | Explain the scientific background and rationale for the investigation being reported | 3 | Recently, a nationwide population-based study demonstrated that metabolic syndrome and each of its diagnostic criteria increase the risk of RVO occurrence. A substantial increase in the prevalence of MetS has been reported in healthy young adults.[9] In addition, a considerable proportion (7.5–26.2%) of CRVO patients were reported to be under 50 years old.[7, 10] Since visual disturbance in young adults may lead to a significant impact on social participation,[11] analyzing the association between MetS and the occurrence of RVO in young patients may have clinical importance. As the incidence of RVO is lower in younger adults, information regarding the risk factors of RVO in the younger population is limited. |
| Objectives | 3 | State specific objectives, including any prespecified hypotheses | 3 | In the present study, we investigated the association between MetS and the risk of RVO development in young Korean patients through a nationwide, large-scale retrospective cohort study based on the Korean National Health Insurance Database. |
| Methods | | | |  |
| Study design | 4 | Present key elements of study design early in the paper | 3-4 |  |
| Setting | 5 | Describe the setting, locations, and relevant dates, including periods of recruitment, exposure, follow-up, and data collection | 4-5 | The data analyzed in this study were derived from the National Health Information Database (NHID), which includes all data from the NHIS covering the entire population of the Republic of Korea. |
| Participants | 6 | (*a*) *Cohort study*—Give the eligibility criteria, and the sources and methods of selection of participants. Describe methods of follow-up  *Case-control study*—Give the eligibility criteria, and the sources and methods of case ascertainment and control selection. Give the rationale for the choice of cases and controls  *Cross-sectional study*—Give the eligibility criteria, and the sources and methods of selection of participants | 4 | A total of 1,571,091 adults aged ≥20 and <40 years who underwent four serial health examinations between January 1, 2009, and December 31, 2012, were selected from the NHID. The database was collected over four consecutive years from the first health examination. Individuals with a history of RVO, missing health examination data or covariates, or with a diagnosis of RVO within one year from the last health examination (index date) were excluded. Ultimately, 1,408,093 adults were included in the analysis. |
|  |  | (*b*) *Cohort study*—For matched studies, give matching criteria and number of exposed and unexposed  *Case-control study*—For matched studies, give matching criteria and the number of controls per case | NA |  |
| Variables | 7 | Clearly define all outcomes, exposures, predictors, potential confounders, and effect modifiers. Give diagnostic criteria, if applicable | 5 |  |
| Data sources/ measurement | 8* | For each variable of interest, give sources of data and details of methods of assessment (measurement). Describe comparability of assessment methods if there is more than one group | *5* | Data are summarized as mean ± standard deviation for continuous variables and number (%) for categorical variables. One-way analysis of variance and the chi-square test were used to evaluate significant differences in baseline characteristics among groups categorized by the number of MetS components. The incidence rate of RVO was computed by dividing new-onset RVO cases by the total follow-up duration and was presented as per 1000 person-years (PY). |
| Bias | 9 | Describe any efforts to address potential sources of bias |  |  |
| Study size | 10 | Explain how the study size was arrived at | 4 | A total of 1,571,091 adults aged ≥20 and <40 years who underwent four serial health examinations between January 1, 2009, and December 31, 2012, were selected from the NHID. The database was collected over four consecutive years from the first health examination. Individuals with a history of RVO, missing health examination data or covariates, or with a diagnosis of RVO within one year from the last health examination (index date) were excluded (Fig. 2). Ultimately, 1,408,093 adults were included in the analysis. |

Continued on next page

| Quantitative variables | 11 | Explain how quantitative variables were handled in the analyses. If applicable, describe which groupings were chosen and why | 4-5 | The MetS was defined using the modified waist circumference criteria of the Korean Society for the Study of Obesity and the guidelines of the National Cholesterol Education Program Third Adult Treatment Panel (NCEP-ATP III) as the presence of ≥3 of the following: increased WC [≥90 cm in males or ≥85 cm in females], elevated TG [≥150 mg/dL (1.7 mmol/L) or drug treatment for elevated TG], low HDL-C [<40 mg/dL (1 mmol/L) in males and <50 mg/dL (1.3 mmol/L) in females or drug treatment for low HDL-C], elevated blood pressure [systolic blood pressure ≥130 mmHg or diastolic blood pressure ≥85 mmHg or current use of antihypertensives], and impaired fasting glucose [fasting plasma glucose ≥100 mg/dL (5.6 mmol/L) or current use of anti-diabetes] [15-17]. At each health examination, the presence of MetS and the number of fulfilled MetS components were calculated. “Metabolic burden” was defined in the following ways during four health examinations: (1) cumulative number of MetS diagnosed at each health examination (0–4 times); and (2) cumulative number of each MetS component diagnosed at each health examination (0–4 times per MetS component). |
| --- | --- | --- | --- | --- |
| Statistical methods | 12 | (*a*) Describe all statistical methods, including those used to control for confounding | 5-6 | Data are summarized as mean ± standard deviation for continuous variables and number (%) for categorical variables. One-way analysis of variance and the chi-square test were used to evaluate significant differences in baseline characteristics among groups categorized by the number of MetS components. The incidence rate of RVO was computed by dividing new-onset RVO cases by the total follow-up duration and was presented as per 1000 person-years (PY). The association between MetS status frequency and RVO incidence was estimated using Cox proportional hazards regression models. The risk of RVO according to the number of patients with MetS compared with the non-MetS group was expressed as HRs with 95% CIs. Model 1 represented an unadjusted risk, and Model 2 was adjusted for age and sex. Model 3 was adjusted for age; sex; smoking status (never smoker, ex-smoker, or current smoker); alcohol intake (non-, mild, or heavy drinker, g/day); regular exercise (performing >30 min of moderate physical activity ≥5 times a week or >20 min of vigorous physical activity ≥3 times a week); and low-income level (income in the lower 20% of the entire Korean population of subjects supported by the medical aid program)[18]. Model 4 was additionally adjusted for systolic blood pressure, fasting glucose, the logarithm of TG, and HDL-C levels. A P value of less than 0.05 was considered statistically significant. Data collection and statistical analysis was performed using SAS version 9.4 (SAS Institute, Cary, NC). |
|  |  | (*b*) Describe any methods used to examine subgroups and interactions | 5 |  |
|  |  | (*c*) Explain how missing data were addressed | 4 |  |
|  |  | (*d*) *Cohort study*—If applicable, explain how loss to follow-up was addressed  *Case-control study*—If applicable, explain how matching of cases and controls was addressed  *Cross-sectional study*—If applicable, describe analytical methods taking account of sampling strategy | 5 | The follow-up period was the time from the index date to the occurrence of RVO or December 31, 2018, whichever occurred first. |
|  |  | (*e*) Describe any sensitivity analyses | NA |  |
| Results | | | | |
| Participants | 13* | (a) Report numbers of individuals at each stage of study—eg numbers potentially eligible, examined for eligibility, confirmed eligible, included in the study, completing follow-up, and analysed | 6 | Table 1 presents the baseline characteristics of 1,408,093 individuals categorized according to the number of MetS diagnoses over four health examinations. |
|  |  | (b) Give reasons for non-participation at each stage | NA |  |
|  |  | (c) Consider use of a flow diagram | 4, 26 |  |
| Descriptive data | 14* | (a) Give characteristics of study participants (eg demographic, clinical, social) and information on exposures and potential confounders | 6-8 | Table 1 presents the baseline characteristics of 1,408,093 individuals categorized according to the number of MetS diagnoses over four health examinations. The mean age of the total study population was 32.45 ± 4.11 years, and 1,011,343 (71.8%) were men. The study participants were classified into five groups according to the number of MetS diagnostic criteria met during four consecutive annual health examinations. The number of individuals included in each group was 1,072,217 (76.1%), 165,760 (11.8%), 80,501 (5.7%), 50,712 (3.6%), and 38,903 (2.8%) (0, 1, 2, 3, and 4 times, respectively). |
|  |  | (b) Indicate number of participants with missing data for each variable of interest | 6-8 |  |
|  |  | (c) *Cohort study*—Summarise follow-up time (eg, average and total amount) | 5 |  |
| Outcome data | 15* | *Cohort study*—Report numbers of outcome events or summary measures over time | *10* |  |
|  |  | *Case-control study—*Report numbers in each exposure category, or summary measures of exposure | *NA* |  |
|  |  | *Cross-sectional study—*Report numbers of outcome events or summary measures | *NA* |  |
| Main results | 16 | (*a*) Give unadjusted estimates and, if applicable, confounder-adjusted estimates and their precision (eg, 95% confidence interval). Make clear which confounders were adjusted for and why they were included | 10 | Table 2 shows the incidence of RVO according to the number of MetS and the number of MetS components during the four health examinations. The cumulative number of MetS diagnoses at each health examination and the risk of RVO showed a positive correlation: adjusted HRs with 95% CIs of groups meeting the diagnostic criteria of MetS 1, 2, 3, and 4 times compared to 0 times were 1.2 (1.03–1.39), 1.35 (1.12–1.63), 1.6 (1.28–1.95), and 1.71 (1.36–2.15), P for trend <0.001). |
|  |  | (*b*) Report category boundaries when continuous variables were categorized | NA |  |
|  |  | (*c*) If relevant, consider translating estimates of relative risk into absolute risk for a meaningful time period | NA |  |

Continued on next page

| Other analyses | 17 | Report other analyses done—eg analyses of subgroups and interactions, and sensitivity analyses | 14-18 | Additional subgroup analyses for RVO development and the cumulative number of MetS were performed and are presented in Table 3. MetS increased the risk of RVO development greater in the female subgroup than in the male subgroup (adjusted HR (95% CI), 4.76 (2.48–9.15) for female, 1.49 (1.17–1.91) for male; P for interaction <0.0001; when diagnosed four times consecutively). Other variables including obesity, current smoking, heavy drinking, and regular performance did not show significant differences in the subgroup analyses. |
| --- | --- | --- | --- | --- |
| Discussion | | | | |
| Key results | 18 | Summarise key results with reference to study objectives | 19 | The key findings of this study can be summarized as follows: (1) the cumulative burden of MetS during the four health examinations had a linear correlation with the risk of RVO, (2) the cumulative burden of each MetS component showed a positive association with the risk of RVO, (3) among the five metabolic components, increased WC had the greatest increase in RVO risks, and (4) MetS increased the risk of RVO greater in female. |
| Limitations | 19 | Discuss limitations of the study, taking into account sources of potential bias or imprecision. Discuss both direction and magnitude of any potential bias |  |  |
| Interpretation | 20 | Give a cautious overall interpretation of results considering objectives, limitations, multiplicity of analyses, results from similar studies, and other relevant evidence | 19-20 |  |
| Generalisability | 21 | Discuss the generalisability (external validity) of the study results | 20 | Given the association found in this study, prompt detection of metabolic derangements and their treatment might be important for decreasing the risk of RVO in young adults, especially in females. |
| Other information | |  | | |
| Funding | 22 | Give the source of funding and the role of the funders for the present study and, if applicable, for the original study on which the present article is based | 21 | This study was supported by Kim’s Eye Hospital Research Center. The funder had no role in study design, data collection and analysis, decision to publish. |

*Give information separately for cases and controls in case-control studies and, if applicable, for exposed and unexposed groups in cohort and cross-sectional studies.

**Note:** An Explanation and Elaboration article discusses each checklist item and gives methodological background and published examples of transparent reporting. The STROBE checklist is best used in conjunction with this article (freely available on the Web sites of PLoS Medicine at http://www.plosmedicine.org/, Annals of Internal Medicine at http://www.annals.org/, and Epidemiology at http://www.epidem.com/). Information on the STROBE Initiative is available at www.strobe-statement.org.
