# Supplementary material for: Cumulative effect of metabolic syndrome on the risk of retinal vein occlusion in young patients: A nationwide population-based study
Source: PLoS One. 2024 May 20;19(5):e0303871. doi: 10.1371/journal.pone.0303871 (PMC11104591; doi:10.1371/journal.pone.0303871)
Supplement: Attachment — Submitted filename: Response to Reviewers.docx [file pone.0303871.s003.docx]

*General comments

Please review your reference list to ensure that it is complete and correct. If you have cited papers that have been retracted, please include the rationale for doing so in the manuscript text, or remove these references and replace them with relevant current references. Any changes to the reference list should be mentioned in the rebuttal letter that accompanies your revised manuscript. If you need to cite a retracted article, indicate the article’s retracted status in the References list and also include a citation and full reference for the retraction notice.

- Response: Thank you for the instructions and comments. I have double-checked the references on the manuscript, and there were no retracted papers found.

Reviewer #1

Please double-check and amend the discrepancy between the methodology section, which states the use of the National Health Insurance Service (NHIS)'s Database, and the acknowledgment and data availability statement, which indicate the use of Health Insurance Review and Assessment Service (HIRA) data.

- Response: Thank you for the comment. I have checked the discrepancy between the methods section and the data availability statement and acknowledgment, and revised the data availability statement and acknowledgment as follows.

(After revision, page 1, line 18)

**Data Availability Statement:** Data are available from the Korea National Health Insurance Sharing Service (https://nhiss.nhis.or.kr/bd/ay/bdaya001iv.do;jsessionid=KiOt6ilaTGXMmR1mbeeA9Ax7nJjIYDsKr4UJAJvHhNyo511E0PovM1WfrGSaegL6.primrose22_servlet_engine1, Tel.: (82) 33-736-2432, 2433) for researchers who meet the criteria for access to confidential data.

(After revision, page 24, line 279)

**Acknowledgments**

The authors wish to thank the National Health Insurance Service for providing the data regarding the original cohort.
